# Idiopathic superior mesenteric venous thrombosis requiring bowel resection: a report of four cases

**DOI:** 10.1186/s40792-024-01916-8

**Published:** 2024-05-14

**Authors:** Kazuto Kamohara, Yoshihiro Miyazaki, Hiromitsu Nakahashi, Kinji Furuya, Manami Doi, Osamu Shimomura, Shinji Hashimoto, Kazuhiro Takahashi, Yohei Owada, Koichi Ogawa, Yusuke Ohara, Yoshimasa Akashi, Tsuyoshi Enomoto, Tatsuya Oda

**Affiliations:** Department of Gastrointestinal and Hepato-Biliary-Pancreatic Surgery, Faculty of Medicine, University of Tsukuba, 1-1-1 Tennodai, Tsukuba , Japan Ibaraki, 305-8575

**Keywords:** Superior mesenteric venous thrombosis, Idiopathic, Bowel resection, Anticoagulation

## Abstract

**Background:**

Superior mesenteric venous thrombosis (SMVT) is mostly treated with anticoagulation therapy; however, SMVT can lead to irreversible bowel ischemia and require bowel resection in the acute or subacute phase.

**Case presentation:**

We report four cases of SMVT that required careful observation and bowel resection. Case 1: A 71-year-old man presented with abdominal pain, diarrhea, and vomiting that showed a completely occluded SMV with thrombus and small bowel ischemia. Case 2: A 47-year-old man presented with abdominal pain, peritoneal irritation symptoms, and a completely occluded SMV with thrombus, ischemia of the small bowel, and massive ascites. Case 3: A 68-year-old man presented with abdominal pain and vomiting for several days and showed a partially occluded SMV with a thrombus, bowel ischemia, and massive ascites. Case 4: A 68-year-old man presented with acute abdominal pain and a partially occluded SMV with thrombus and bowel edema without ischemic changes. Anticoagulation therapy was administered; however, 3 days later, abdominal pain and bowel ischemia worsened. Bowel resection was performed in all cases.

**Conclusions:**

Most idiopathic SMVT cases can be treated with anticoagulation therapy or endovascular thrombectomy. However, in cases with peritoneal irritation signs, these treatments may be ineffective, and bowel resection may be required.

**Supplementary Information:**

The online version contains supplementary material available at 10.1186/s40792-024-01916-8.

## Background

Mesenteric venous thrombosis (MVT) often presents as acute abdomen. MVT represents 6–9% of all cases of acute mesenteric ischemia [1]. SMVT is caused by hypercoagulable states, such as protein C and antithrombin deficiency; local vessel injury, such as inflammation, infection, and intra-abdominal malignancy; and venous stasis, such as heart failure and cirrhosis [2]. Most cases of SMVT with obvious risk factors are treated with anticoagulation therapy, but idiopathic SMVT without risk factors can lead to irreversible bowel ischemia and require bowel resection. It progresses rapidly and requires prompt and accurate treatment. We must be vigilant of irreversible bowel necrosis signs, such as those of peritoneal irritation. In this report, we describe four cases of acute idiopathic SMVT treated with bowel resection because of rapid progression.

## Case presentation

### Case 1

**Fig. 1:**
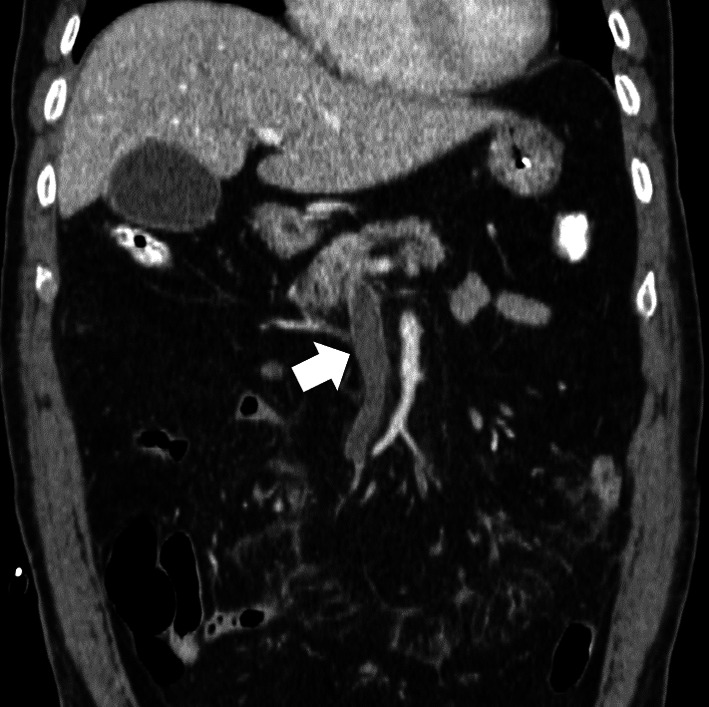
Contrast enhancement computed tomography (CE-CT) findings (coronal view) of Case 1. SMV is completely occluded with a thrombus (arrow). SMV, superior mesenteric vein

A 71-year-old man presented at a local hospital with abdominal pain, diarrhea, and vomiting for 8 h. The patient was referred to our hospital with SMVT. The patient had a history of hypertension and diabetes mellitus. The vital signs were as follows: heart rate 115 bpm, blood pressure 90/55 mmHg, SpO_2_ 94%, and body temperature 38.2 °C. The patient had signs of peritoneal irritation. Laboratory examination data were as follows: leukocyte count, 16.4 × 10^3^/μL; C-reactive protein (CRP), 3.7 mg/dL; D-dimer, 24.3 μg/mL; and lactate, 8.3 mmol/L. Contrast-enhanced computed tomography (CE-CT) revealed a completely occluded SMV with a thrombus (Fig. 1a) and partially occluded portal and splenic veins, and most of the small bowel wall presented poor contrast enhancement and massive ascites. The mean CT value of the ischemic bowel wall layer was 43.2/47.9 HU (non-contrast/portal phase CT). The patient was diagnosed with irreversible bowel ischemia caused by SMVT, and bowel resection was performed immediately. Moderate hemorrhagic ascites and a gangrenous small bowel measuring 350 cm were found (Fig. 1b). The bowel was then resected and anastomosed. A catheter was placed from the ileocecal vein to the SMV for postoperative local anticoagulation. The tip of the catheter was placed at the SMV confluence. The catheter was removed after fistulization of the mesentery and abdominal wall at the site of catheter insertion. The operation time was 283 min, and the blood loss was 2100 mL, including bloody ascites. The postoperative course was uneventful. We initiated anticoagulation therapy with intravenous heparin and urokinase through the catheter in the SMV on postoperative day (POD) 1. Oral solid food intake was initiated on POD 15. Intravenous anticoagulation therapy was switched to an oral vitamin K antagonist. The patient was discharged on POD 30, and no recurrence was found on the follow-up CT for 3 years.

### Case 2

**Fig. 2:**
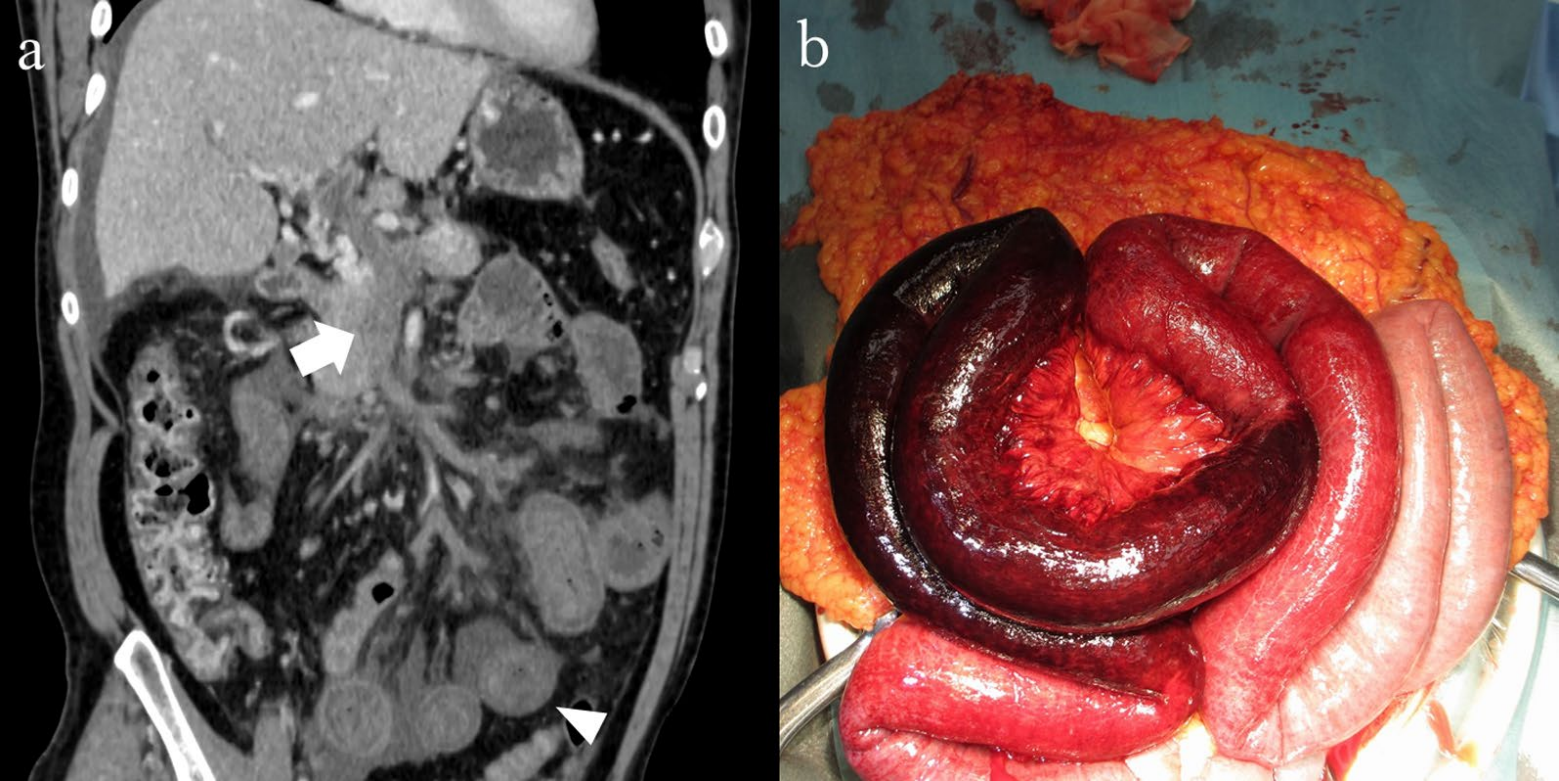
**a** CE-CT findings (coronal view) of Case 2. Thrombus completely occludes SMV and portal vein (arrow). Jejunal wall with poor contrast enhancement and massive ascites are observed (arrowhead). **b** Intraoperative findings. A gangrenous small bowel measuring 160 cm is resected

A 47-year-old man presented to a local hospital with abdominal pain for 10 days. CE-CT revealed a completely occluded SMV with a thrombus, ischemia of the small bowel, and massive ascites. The patient was referred to our hospital with SMVT. The patient had a history of psoriasis vulgaris. Vital signs were normal; however, the patient presented with symptoms of peritoneal irritation. Laboratory examination data were as follows: leukocyte count, 16.1 × 10^3^/μg; CRP, 16.9 mg/dL; D-dimer, 9.6 μg/mL; and lactate, 1.0 mmol/L. CE-CT revealed a completely occluded SMV and portal vein with a thrombus (Fig. 2a), with most jejunal walls having poor contrast enhancement and massive ascites. The mean CT value of the ischemic bowel wall layer was 47.3/47.6 HU (non-contrast/portal phase CT). The patient was diagnosed with irreversible bowel ischemia caused by SMVT, and bowel resection was performed immediately. Moderate hemorrhagic ascites and a gangrenous small bowel measuring 160 cm were found (Fig. 2b). The bowel was then resected and anastomosed. The operation time was 131 min, and blood loss was 715 mL, including bloody ascites. The postoperative course was uneventful. Anticoagulation therapy with intravenous heparin was initiated on POD 2. Intravenous anticoagulation therapy was switched to an oral vitamin K antagonist on POD 7. Oral intake of solid food was initiated on POD 10. The patient was discharged on POD 23 and showed no recurrence on the follow-up CT for 2 years.

### Case 3

**Fig. 3:**
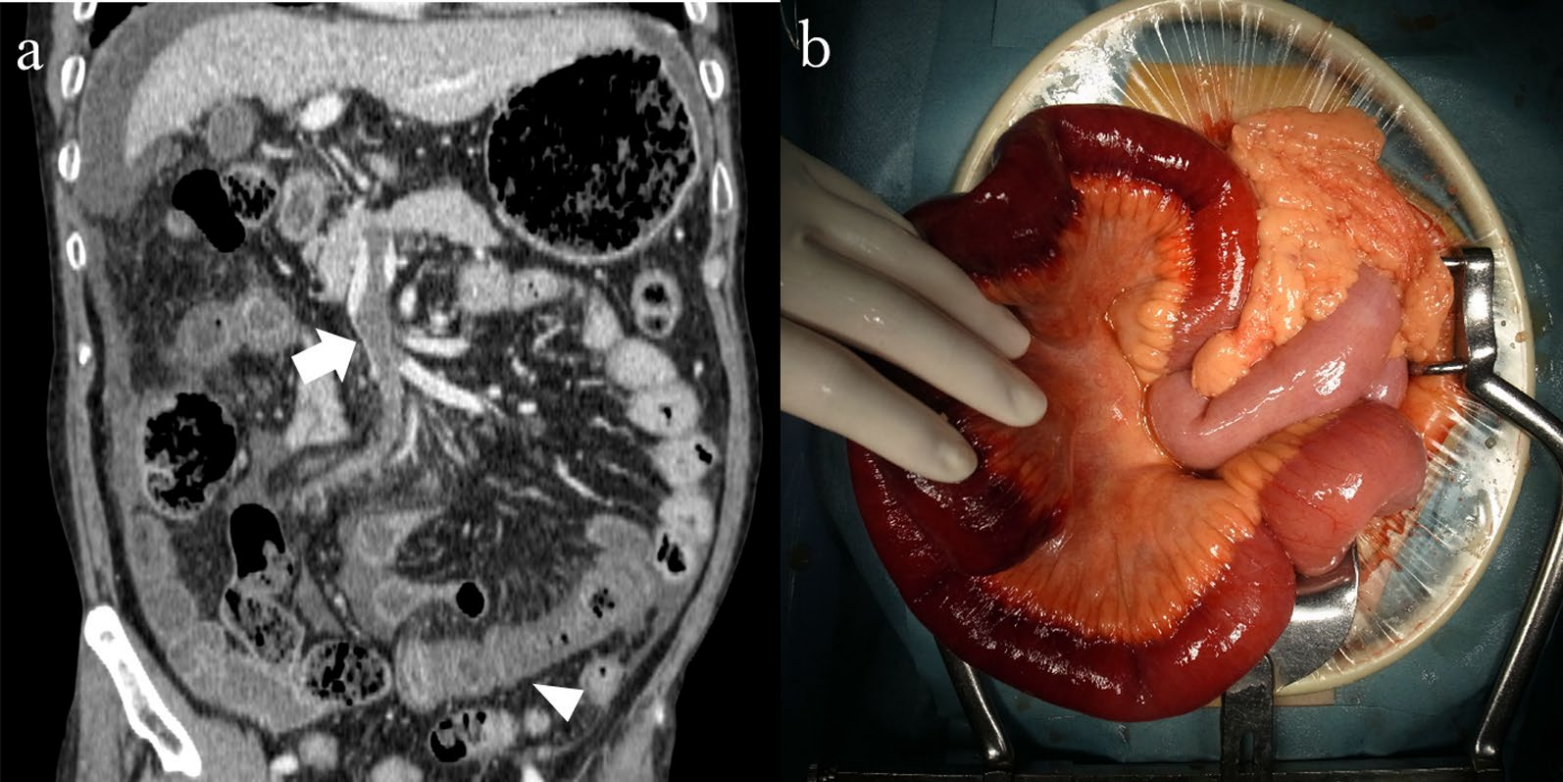
**a** CE-CT findings (coronal view) of Case 3. Thrombus partially occludes SMV (arrow). Small bowel wall with poor contrast enhancement and massive ascites are observed (arrowhead). **b** Intraoperative findings. A gangrenous small bowel measuring 65 cm is resected

A 68-year-old man presented with several days of abdominal pain and vomiting and was referred to our hospital for emergency care. The patient had a history of microscopic polyangiitis, idiopathic thrombocytopenic purpura, interstitial pneumonia, and diabetes mellitus. The patient had normal vital signs but presented with symptoms of peritoneal irritation. Laboratory examination data were as follows: leukocyte count, 26.8 × 10^3^/μg; CRP, 0.04 mg/dL; D-dimer, 9.6 μg/mL; and lactate, 2.7 mmol/L. CE-CT revealed a partially occluded SMV with a thrombus (Fig. 3a), a fully patent portal vein, and an ileal wall at short range with poor contrast enhancement and massive ascites. The mean CT value of the ischemic bowel wall layer was 16.7/28.0 HU (non-contrast/portal phase CT). The patient was diagnosed with irreversible bowel ischemia caused by SMVT, and bowel resection was performed immediately. Moderate hemorrhagic ascites and a gangrenous small bowel measuring 65 cm were found (Fig. 3b). The bowel was resected. Stoma placement was performed instead of anastomosis because of the patient’s comorbidities that required high-dose steroid treatment. The operation time was 322 min, and the blood loss was 1990 mL, including bloody ascites. Anticoagulation therapy with intravenous heparin was initiated on POD 2. Intravenous anticoagulation therapy was switched to an oral vitamin K antagonist on POD 7. Stoma closure was performed on POD 30. The patient died of interstitial pneumonia 74 days after the initial surgery.

### Case 4

**Fig. 4:**
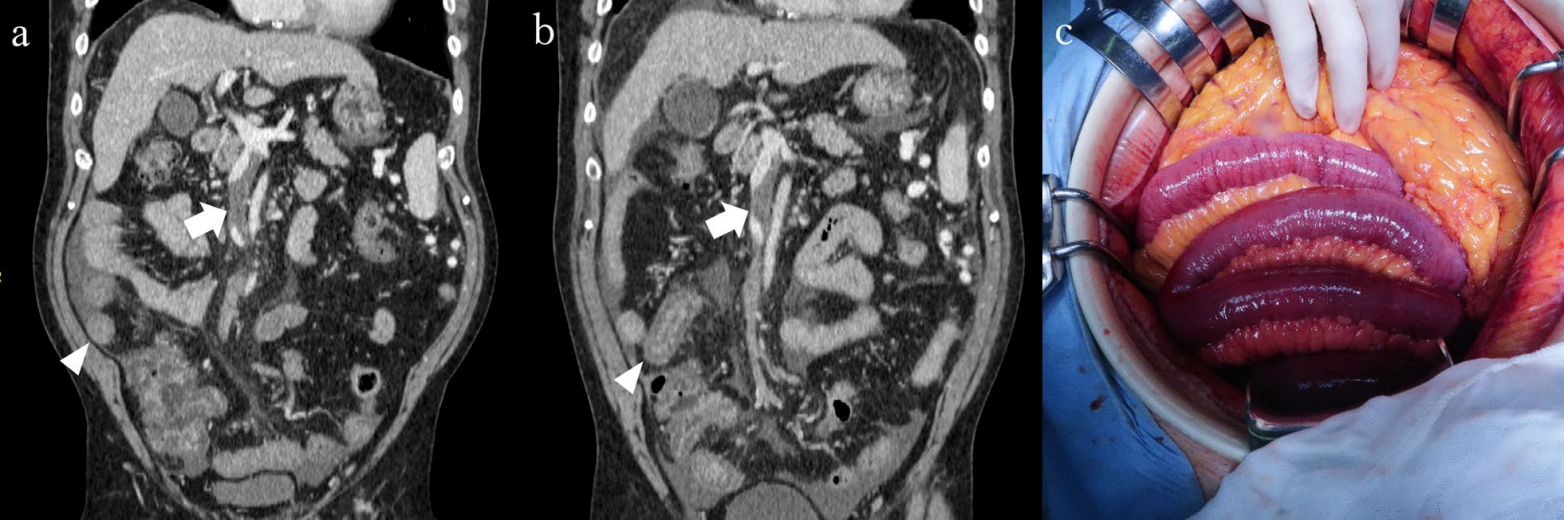
**a** CE-CT findings on admission (coronal view) of Case 4. Thrombus occludes the SMV but not the splenic vein (arrow). Ileum edema at short range and moderate ascites are observed (arrowhead). **b** CE-CT findings at the time of worsening symptoms (coronal view) of Case 4. The thrombus in SMV has not changed (arrow). Ileum edema is worsening (arrowhead). **c** Intraoperative findings. A gangrenous small bowel measuring 50 cm is resected

A 68-year-old man presented to a local hospital with acute abdominal pain for several hours. The patient was referred to our hospital for further evaluation. The patient had a history of angina pectoris and diabetes mellitus. The patient had normal vital signs and no peritoneal irritation symptoms. Laboratory examination was as follows: leukocyte count, 9.0 × 10^3^/μg; CRP, 4.4 mg/dL; D-dimer, 26.0 μg/mL; and lactate, 2.3 mmol/L. CE-CT revealed a partially occluded SMV with a thrombus (Fig. 4a), a fully patent portal vein, and a small area of ileal edema and ascites accumulation but no intestinal ischemia. The mean CT value of the ischemic bowel wall layer was 42.3/75.1 HU (non-contrast/portal phase CT). Anticoagulation therapy with intravenous heparin (250U/kg dose) was administered. Three days later, the patient developed a fever, and the abdominal pain worsened. However, the patient had no symptoms of peritoneal irritation. CE-CT showed increased ascites and worsening of the small bowel edema (Fig. 4b). The mean CT value of the ischemic bowel wall layer was 38.2/40.5 HU (non-contrast/portal phase CT). Anticoagulation treatment was judged to have failed, and emergency small bowel resection was performed. Moderate hemorrhagic ascites and a gangrenous small bowel measuring 50 cm were found (Fig. 4c). The ischemic bowel was then resected. We attempted to remove the SMV thrombus but could not complete it because of excessive bleeding. The operation time was 394 min, and the blood loss was 3350 mL, including bloody ascites. Anticoagulation therapy was initiated on POD 1. Oral solid food intake was initiated on POD 12. Anticoagulation therapy was switched to oral edoxaban on POD 20. The patient was discharged on POD 47, and no recurrence was found on follow-up CT for 2 years.

## Discussion

We have reported four cases of idiopathic SMVT that required bowel resection. Most cases of SMVT without peritoneal irritability and/or unstable general condition are primarily treated with anticoagulation therapy. However, in approximately 20% of cases with peritoneal irritation signs, anticoagulation therapy may be ineffective, and bowel resection may be necessary [12]. One of the four patients needed bowel resection because of disease progression during anticoagulation therapy (Table 1). In the acute or subacute phase, SMVT can progress rapidly and require bowel resection. This study makes a novel contribution to the literature in that it draws attention to the importance of careful monitoring of SMVT.

**Table 1:** Characteristics of patients with superior mesenteric venous thrombosis

Case	Age	Sex	Past medical history	Symptoms	Time from the onset to the operation	Peritoneal irritation	CT findings	CT value of ischemic bowel wall (non-contrast/portal phase)	Preoperative anticoagulation therapy	Bowel resection/ anastomosis	Postoperative anticoagulation therapy
1	71	Male	Hypertension, polycythemia, diabetes mellitus	Abdominal pain, diarrhea, vomiting	8 h	+	SMV obstruction by thrombosis	43.2/47.9 HU	–	+ / +	Urokinase by catheter, Heparin → VKA
2	47	Male	Psoriasis vulgaris	Abdominal pain	10 days	+	PV and SMV obstruction by thrombosis	47.3/47.6 HU	–	+ / +	Heparin → VKA
3	68	Male	Microscopic polyangiitis, interstitial pneumonia, diabetes mellitus	Abdominal pain	Several days	+	SMV obstruction by thrombosis	16.7/28 HU	–	±	Heparin → VKA
4	68	Male	Angina, diabetes mellitus	Abdominal pain	3 days	–	SMV obstruction by thrombosis	42.3/75.1 HU → 38.2/40.5 HU	Heparin	+ / +	Heparin → DOAC^||^

CT, computed tomography; SMV, superior mesenteric vein; PV, portal vein; VKA, vitamin K antagonist; DOAC, direct oral anticoagulant

Although advances in CT and other equipment have enabled early and accurate diagnosis, MVT remains a fatal disease with a mortality rate of 26.8% [3], and readmission after initial treatment has been reported in 47.1% of cases [4]. MVT can be caused by various factors, with approximately 1/3 of patients with MVT having multiple risk factors. Patients with MVT can be treated with anticoagulation therapy. Idiopathic MVT accounts for 20% of patients [1]. All four patients in this study had idiopathic MVT without risk factors. The treatment goal is to prevent bowel ischemia, portal hypertension, exacerbation of thrombosis, and relapse. If the disease is non-idiopathic, the priority is to treat the cause. Even in secondary SMVT, bowel resection must be performed if there is bowel necrosis. Systemic anticoagulation is the initial treatment of choice in most cases. It must be started as early as possible to prevent progression to irreversible bowel necrosis. Anticoagulation treatment alone may be sufficient in mild cases; however, in anticoagulant-resistant patients, endovascular therapy or bowel resection is necessary [5]. In Case 4, although anticoagulation therapy with heparin was chosen as the initial treatment, the disease progressed, and the bowel ischemia observed in the CE-CT expanded; therefore, bowel resection became necessary.

Endovascular therapy, which includes mechanical thrombectomy and local thrombolysis, is minimally invasive and can avoid bowel resection [6–8]. However, owing to the small number of cases, it is unclear whether endovascular thrombectomy can avoid bowel resection in all SMVT cases, as it is ineffective in patients with irreversible bowel ischemia. In addition, even if systemic anticoagulation or endovascular therapy works in the acute phase, bowel stenosis can occur several months later in some patients [5, 9]. It has been reported that 55% of patients require bowel resection after endovascular therapy for acute venous thrombosis because of bowel necrosis or stenosis [10].

There are no clear criteria for performing bowel resection on patients with SMVT. The pathogenesis of SMVT is considered arterial ischemia due to blood flow congestion in the mesenteric veins. Several studies have reported the risk factors for bowel resection in SMVT, including leukocytosis [8], peritonitis, lactic acidosis, and bowel wall thickening on CT scans [10]. In all cases, CT values were useful for detecting ischemic change. CT values and the ratio of non-contrast CT to portal phase CT are shown in Table 2. In Case 4, the CT value ratio of non-contrast CT to portal phase CT decreased, indicating irreversible ischemic changes had occurred. A report suggests that a CT value ratio of the bowel (enhanced/unenhanced image) lower than 1.5 is useful in diagnosing strangulated ileus [11]. However, it is often difficult to determine whether irreversible bowel ischemia has occurred preoperatively. There is a time lag between onset and ischemia. However, as shown in the present cases, it varies by case (8 h to 10 days), and little is known in the literature. Onset is non-specific with abdominal pain, nausea and vomiting, etc. If they are, they may not receive a full work-up, and SMVT may not be diagnosed. In Cases 1–3, the patients presented with rapidly progressive symptoms and peritoneal irritation signs on admission and were diagnosed with irreversible ischemic changes. The diagnosis of peritoneal irritation was made by at least three surgeons, including the board certificated surgeon. Laparoscopic diagnosis was considered inappropriate because of poor working space due to a dilated bowel. Therefore, we performed laparotomy in all four cases. Several studies have proposed treatment protocols for SMVT, and most of them have suggested that bowel resection should be performed in patients with peritoneal irritation signs [5, 10]. Even if the disease is mild at the time of initial diagnosis, it should be carefully monitored for progression. In our four cases, peritoneal irritation signs were useful as an indicator. A previous report showed that approximately 85% of patients with SMVT could be treated with systemic anticoagulation alone, 5% could avoid bowel resection with endovascular treatment, and 10% required bowel resection [5].

## Conclusions

SMVT with signs of peritoneal irritation may require bowel resection and must be carefully observed.

### Supplementary Information

Supplementary file 1. CT values of bowel whole layer (HU).

## Data Availability

No additional data.

## Abbreviations

CE-CT: Contrast-enhanced computed tomography
CRP: C-reactive protein
MVT: Mesenteric venous thrombosis
POD: Postoperative day
SMVT: Superior mesenteric venous thrombosis
