# Supplementary material for: Idiopathic superior mesenteric venous thrombosis requiring bowel resection: a report of four cases
Source: Surg Case Rep. 2024 May 14;10:122. doi: 10.1186/s40792-024-01916-8 (PMC11093935; doi:10.1186/s40792-024-01916-8)
Supplement: Supplementary file 1 — CT values of bowel whole layer (HU). [file 40792_2024_1916_MOESM1_ESM.docx]

|  | | | | | | |
| --- | --- | --- | --- | --- | --- | --- |
|  | Normal bowel | | | Ischemic bowel | | |
|  | Non-contrast CT | Portal phase CT | Ratio | Non-contrast CT | Portal phase CT | Ratio |
| Case 1 | 47.6 | 85 | 1.79 | 43.2 | 47.9 | 1.11 |
| Case 2 | 44.2 | 73.2 | 1.66 | 47.3 | 47.6 | 1.01 |
| Case 3 | 29.5 | 108.2 | 3.67 | 16.7 | 28 | 1.68 |
| Case 4 (on admission) | 48.8 | 102.9 | 2.11 | 42.3 | 75.1 | 1.78 |
| Case 4 (3 days later) | 44.6 | 90.1 | 2.02 | 38.2 | 40.5 | 1.06 |

**Supplementary 1** CT values of bowel whole layer (HU)
